# Successful Regenerative Endodontic Procedure of a Nonvital Immature Permanent Central Incisor Using Amniotic Membrane as a Novel Scaffold

**DOI:** 10.3390/dj6030036

**Published:** 2018-08-02

**Authors:** Nandini Suresh, Buvaneshwari Arul, Dinesh Kowsky, Velmurugan Natanasabapathy

**Affiliations:** Department of Conservative Dentistry and Endodontics, Faculty of Dental Sciences, Meenakshi Academy of Higher Education and Research (MAHER), Alapakkam Main Road, Maduravoyal 600095, India; buvana.ak@gmail.com (B.A.); kowskydinesh@gmail.com (D.K.); vel9911@yahoo.com (V.N.)

**Keywords:** amniotic membrane, cone beam computed tomography, regenerative endodontic procedure, scaffolds

## Abstract

Successful regenerative endodontic procedure was performed in nonvital immature permanent central incisor (Stage-4 root development) using human amniotic membrane (HAM) as a novel scaffold. The treatment was performed according to the American Association of Endodontics guidelines with minimal canal instrumentation, 1% Sodium hypochlorite as irrigant and calcium hydroxide as intracanal medicament. During the second appointment, HAM was placed as a scaffold and Biodentine™ was layered over the HAM with glass ionomer cement and resin composite as coronal seal. Preoperative and post-operative cone beam computed tomography (at three years) was taken to assess the treatment outcome. The resolution of disease process and increase in canal width, as well as positive response to pulp sensitivity tests, were observed by the end of three years. There was approximately 78–86% reduction in the volume of periapical lesion size. This case report confirms that HAM can be used as a scaffold material for successful regenerative endodontic procedure (REP).

## 1. Introduction

The paradigm shift towards regenerative endodontic procedures (REP) as an alternative to conventional endodontic therapy for immature necrotic teeth has created a new epoch in endodontics. The successful outcome of REP depends on the basic principles of tissue engineering, the stem cells, signaling molecules and three-dimensional physical scaffold [1]. Despite the meritorious results of REP in restoring the immunological and hemostasis insufficiency and as well as regenerating the pulp dentin complex, the clinical translation of this technique has encountered major setbacks [2]. The two major issues that form the road blocks are the type of stem cells and scaffold that should be used for pulp dentin regeneration [2].

Dental pulp stem cells are indispensable for pulp regeneration. Various natural and synthetic scaffolds are utilized for REP. The clinical protocol for REP is highly variable and has evolved from inducing a blood clot to the placing of platelet rich plasma/fibrin or a collagen sponge as scaffold [3]. Though these techniques showed clinical success, there were inconsistencies in treatment outcomes, which depend on a multitude of variable factors. To overcome these shortcomings, cell transplantation and cell homing techniques were developed. An injectable or casted scaffold is used for these techniques, which acts as a carrier for biomolecules and its controlled release, enhances migration, adhesion and proliferation of stem cells as well as being biodegradable [4]. Despite the scientific validity, both of these techniques have disadvantages. Immunorejection, contamination, and potential loss of cells during storage and economic factors are a few of the barriers encountered by the cell transplantation technique. Though a cell homing technique has proven to completely regenerate the pulp in root canal space, the issue of shortage of stem cells in a large periapical defect must be considered [2].

Human amniotic membrane (HAM), a placental based scaffold has been extensively used in the medical field due to its cell proliferation and regeneration ability. HAM constitutes the innermost layer of placenta, composed of a single epithelial layer, thick basement membrane and avascular stroma [5]. HAM is extensively used in the reconstruction of ocular surface, skin reconstruction, endothelial cell cultivation, local drug delivery as well as graft material after vestibuloblasty [6]. Though HAM has been used in the medical field since 1910, current research has expanded its use in the field of tissue engineering due its ability to enhance epithelization, antiscarring and anti-inflammatory effects. The presence of collagen, elastin, laminin and fibronectin makes this membrane an excellent scaffold in tissue engineering. Contrasting to the other barrier membranes, HAM is biologically active due to the presence of growth factors (GF) that aids cell migration and facilitates wound healing [7]. The advantage of HAM as a scaffold is its lack of immunogenicity and irradiation to remove any potential contamination [8].

Over the recent years, versatile use of HAM has been observed in the field of dentistry in treating periodontal osseous defects [9], coverage of gingival recession, furcation defects as well as ridge preservation [10]. Preserved HAM is found to be a safe biomaterial that aids in the oral and maxillofacial wound healing. HAM along with freeze dried bone allograft reduces pocket depth and improves bone healing in chronic periodontitis patients with grade II furcation defects [11]. Amniotic membrane has been used clinically for guided tissue regeneration in endodontic surgery to restore the attachment apparatus of the tooth [12]. Recent research has brought to light that HAM can be used as a cell delivery vehicle in tissue engineering. Autologous/allogenic cell transfer, attachment and proliferation are supported by the epithelial and stromal sides of HAM. Chen et al. has demonstrated that HAM as a scaffold modulates the environment for human dental apical cells to show osteogenic differentiation. They also have proposed that acellular HAM matrix could be used in bone/tooth regeneration [13].

Inferring from this evidence, it was hypothesized that HAM can be used as a natural scaffold for REP. Its properties could be harnessed for enhancing the outcome of this procedure. This case report presents a progressively healing regenerative procedure in a necrotic immature central incisor by using HAM as a novel scaffold material with a follow up of three years.

## 2. Case Report

**Figure 1:**
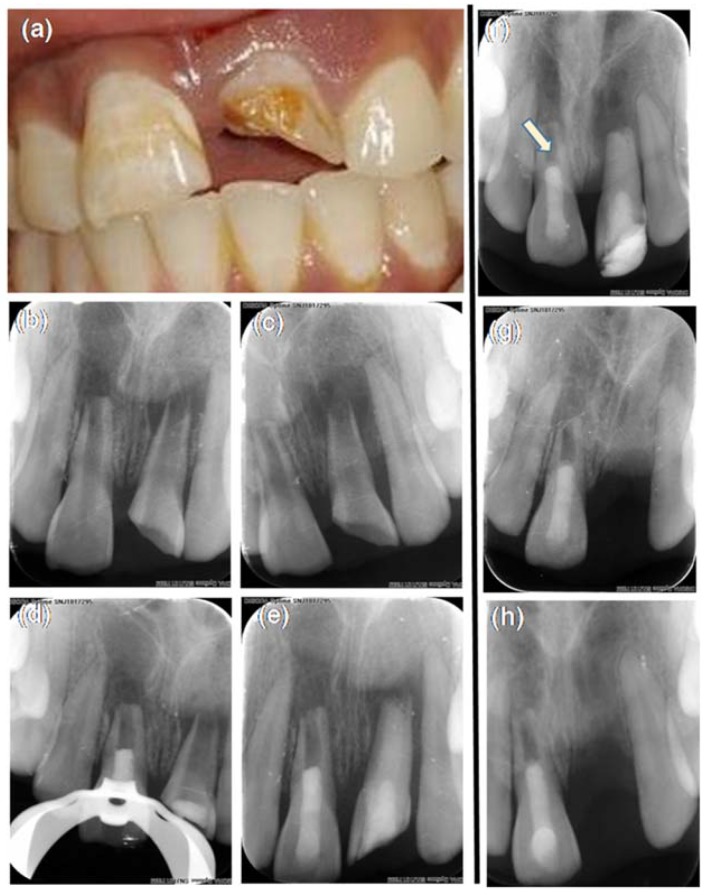
Intraoral periapical radiograph (IOPA) images of preoperative and a three-year follow up of REP in 11. (**a**) pre-operative photograph of maxillary central and lateral incisors; (**b**,**c**) pre-operative IOPA of teeth 11 and 21; (**d**) IOPA showing a coronal seal after placement of HAM in 11; (**e**) immediate postoperative IOPA of 11; (**f**) IOPA at a three-month follow up. The arrow is pointing to the calcific barrier above the Biodentine; (**g**) 19 month follow up IOPA; (**h**) three-year follow up IOPA.

**Figure 2:**
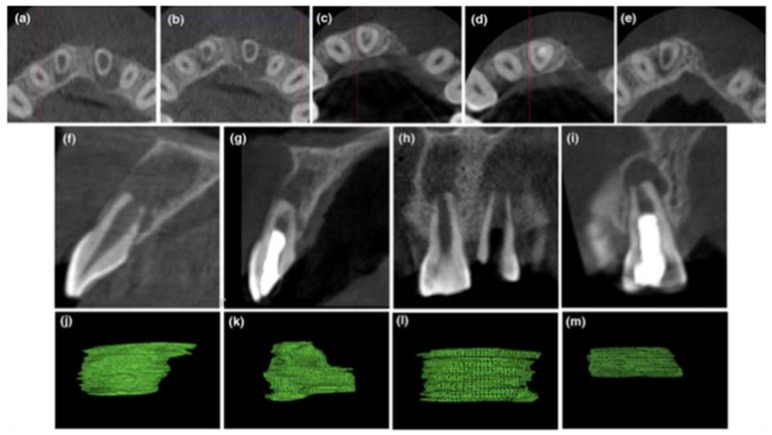
CBCT images of preoperative and three-year follow up of REP in 11. (**a**,**b**) pre-operative axial view revealing root canal dentin thickness and periapical radiolucency at midroot level (**a**) and apical section (**b**); (**c**–**e**) post-operative axial view in the middle and apical region revealing a reduction in size of periapical radiolucency, thickening of root canal wall and mineralized layer above the Biodentine (**e**); (**f**,**g**) pre-operative and post-operative sagittal view showing a reduction in periapical radiolucency; (**h**,**i**) pre-operative and post-operative coronal view showing a reduction in periapical radiolucency and a thickening of the root canal wall; (**j**,**k**) pre-operative and post-operative axial images showing a reduction in volume of periapical lesion by 86% (measured using Volux—Horos viewer for Mac (V2.0.2); (**l**,**m**) pre-operative and post-operative sagittal images showing a reduction in volume of periapical lesion by 78% (measured using Volux—Horos viewer for Mac (V2.0.2).

An 18 year-old female patient reported to our dental outpatient unit with a fractured and discolored tooth in the maxillary anterior region three years before. The patient gave a history of trauma five years back with occasional pus discharge from the gingiva in relation to maxillary right and left central incisor tooth #11 and tooth #21. Medical history was noncontributory. Clinical examination revealed an enamel fracture in relation to tooth #11 and a discolored tooth with enamel–dentin–pulp fracture in relation to tooth #21 (Figure 1a). As the prognosis of tooth #21 was guarded and the regenerative procedure was attempted in tooth #11, this case report will further describe about tooth #11 only. The tooth was not tender to palpation and percussion tests. Sensitivity tests with cold and electric pulp testing was negative. There was no evidence of swelling or a sinus tract. The mobility was within the physiological limits. Radiographic evaluation of tooth #11 revealed an immature apex with thinned out root dentin near the apex of the root with periapical radiolucency (Figure 1b,c). A cone beam computed tomography (CBCT) Promax 3D (Planmeca, Helsinki, Finland) with a limited field of view (FOV) of 3 × 3 cm was taken to aid in diagnosis and treatment planning. By using an inbuilt software program (Planmeca Romexis software V3.5.1, Planmeca, Helsinki, Finland), various linear dimensions were recorded in millimeters. The root length was 7.4 mm. The root dentin thickness in the axial section measured 1.2 mm labially, 0.8 mm mesially, 0.8 mm lingually, and 0.8 mm distally, respectively (Figure 2a,b). The volume of periapical lesion was measured using a Volux–Horos viewer for Mac (V2.0.2, Nimble Co., LLC d/p/a Purview, Annapolis, MD, USA), and a CBCT periapical index (CBCTPAI) score of 5D was assigned to tooth #11 (Figure 2f,h,j,l). A diagnosis of an immature tooth with pulp necrosis and asymptomatic apical periodontitis in relation to tooth #11 arrived.

A regenerative procedure was planned for tooth #11 using HAM as scaffold. The patient was explained about the extensive use of HAM in clinical scenarios [10] and its use in a root canal is off-label. Written informed consent was obtained from the patient after a thorough explanation of treatment procedure. Tooth #11 was anesthetized with 2% lidocaine with adrenaline (Xylocaine, AstraZenecaPharma India Pvt. Ltd., Bangalore, India) and isolated with rubber dam (Dental Dam, Coltène Whaledent, Langenau, Germany). The tooth #11 was accessed with round bur (Dia-burs, Mani Inc., Tochigi, Japan). The working length was determined by electronic apex locator (Root ZX; J Morita MFQ, Kyoto, Japan) and confirmed by taking a digital periapical radiograph (Satelec India Pvt Ltd., Acteon Group, the Hague, the Netherlands). Minimal instrumentation of the root canal was performed by circumferential filing with #80 K file in relation to #11. The canals were gently irrigated with 20 mL of 1% sodium hypochlorite (NaOCl) (Parcan; Septodont, Saint-Maur, France) using EndoVac (Discus Dental, Culver City, CA, USA). The canal was dried with sterile absorbent points (Dentsply Maillefer, Baillaigues, Switzerland). The interappointment medication of calcium hydroxide (Prime Dental products, Mumbai, India) was placed inside the canal and then temporized with cavit (3 M, St Paul, MN, USA).

The patient was recalled after four weeks and was asymptomatic in relation to #11. The access cavity was reopened under rubber dam isolation. Calcium hydroxide was removed with saline using passive ultrasonic irrigation (Satelec, Acteon group, Merignac, France). Final disinfection was performed with 20 mL of 1% NaOCl and 20 mL of 17% ethylenediamine tetraacetic acid (EDTA) (Dent Wash, Prime Dental products, Thane, India) using EndoVac. The canal was dried with sterile absorbent points. The commercially available processed freeze-dried irradiated human amniotic membrane (ACTREC, Tata memorial hospital tissue bank, Mumbai, India) was cut into small pieces and moistened with saline. The membrane was packed inside the canal incrementally with finger plugger in the apical portion of the canal. ACTREC is the first tissue bank in India to use radiation for the sterilization of biological tissues with an ISO 9001:2000 Certified Quality Management System. The processing of amnion tissue in Tata Memorial Hospital is carried out according to the guidelines of the American Association of Tissue Banks [14]. Biodentine^TM^ (Septodont, Saint-Maur, France) was then placed in the coronal third of the root canal and the access was sealed with glass ionomer cement (Fuji IX, GC, Tokyo, Japan) and resin composite (Figure 1d,e).

The patient was asymptomatic when reviewed after 15 days in relation to tooth #11. At the three-month follow up, the patient was asymptomatic with no signs of swelling or sinus tracts in relation to #11 (Figure 1f). The patient was lost to follow up and returned to the clinic at 19 months. The patient was assessed at 19 months (Figure 1g) and at 36 months (Figure 1h) wherein the patient continued to be asymptomatic in relation to tooth #11. The patient responded to electric pulp vitality and cold testing methods, which was reproducible multiple times. The intra oral radiographic examination revealed healing of periapical lesion, thickening of dentinal walls as well as a mineralized dentin bridge formation over Biodentine™ (Figure 1f).

A postoperative CBCT was performed for the evaluation of treatment outcome. The post-operative reduction of volume of periapical lesion was assessed using a Volux—Horos viewer for Mac (V2.0.2). The CBCT images of #11 revealed a reduction in size of periapical radiolucency, a thickening of the root canal wall and deposition of the mineralized layer in close approximation to Biodentine™ (Figure 2c,d,e,g,i,k,m).

## 3. Discussion

One of the important factors that determine the nature of wound healing is the chronicity of the prior infection. Long-term infection with a large periapical lesion may cause a reduction in the availability of stem cells near the apical area and also can alter the dentin substrate, which will affect the cell attachment and cell proliferation. The presence of bacteria in the biofilms integrated into dentin can mobilize the immune system and result in inflammation, which can ultimately hinder the cell proliferation. Thorough disinfection of the root canal creates a stem cell conducive microenvironment [15]. A striking heterogeneity has been observed in endodontic literature for the protocol used for dentin disinfection during REP. The clinical success is found to range from 85–100% even with varied disinfection protocol [16]. Disinfection protocol was performed in #11 following the American Association of Endodontics (AAE) guidelines [17]. Calcium hydroxide was used as an intracanal medicament as it promotes stem cells of apical papilla survival, proliferation [18] and also stimulates the release of transforming growth factor-β1 (TGF) from dentin [19].

The choice of an appropriate scaffold is critical for the successful outcome of this procedure. In this patient, bleeding was inadequate in the root canal after introducing the file beyond the apex to induce bleeding. Therefore, HAM was used as a scaffold directly without an induction of bleeding in tooth #11. The clinical success encountered with HAM as a scaffold for healing has been mainly attributed to its thick basement membrane and the abundant growth factors present within the membrane. The matrix of basement membrane of HAM contains fibronectin, collagen fibers and other proteoglycans, which provides important biochemical cues for cell adhesion and proliferation, and thus favors tissue reconstruction and remodeling [20]. Although preservation procedures like freeze-drying and irradiation partially destroyed the amniotic epithelium, the continuity of the basement membrane remains intact [21], retaining most of the characteristics similar to that of fresh HAM. Growth factors are essential for the regulation of cellular processes including growth, proliferation and differentiation. The vascular endothelial growth factor and hepatocyte growth factor secreted by HAM maintains a balance between TGF-β1 and TGF-β3, which prevents scarring (repair) and hastens regeneration [22].

Various studies have proven that preserved HAM is viable and aids in healing and regeneration. Allen et al. reported that the epidermal growth factor (EGF) and transforming growth factor (TGF)-β1 retention profile in preserved dried HAM is approximately 61% and 86%. The concentration of EGF and TGF-β1 in dried HAM did not change even after 60 weeks of storage (1.2 ng/mg) [23]. It has been proven that tissue inhibitor of metalloproteinases (TIMP)-1 (which modulates matrix metalloproteinases (MMP)) is expressed from freeze-dried HAM even after 12 months [24]. Thus, freeze dried HAM was used as a scaffold in this worst-case scenario, as its outcome would prove the actual competence of this material. Biodentine™ was used to seal the canal as it has shorter setting time, better handling of characteristics and demonstrates osteoinductivity [25].

AAE/American academy of oral and maxillofacial radiology (AAOMR) Joint Position Statement guidelines recommend that, in the absence of clinical signs and symptoms, if limited FOV CBCT was the imaging modality at the time for evaluation and treatment, CBCT can be advised for the follow-up assessment. In the case of REP, the primary clinical outcome must be patient centered, which constitutes healing of diseased tissue, prevention of relapse and well-being of the patient. Continued root development is considered as a secondary treatment outcome, whereas observing a positive response to pulp vitality testing is considered as a tertiary treatment outcome [26]. In our case report, with the use of HAM as a scaffold for REP, we have observed a positive primary (78–86%) as well as successful secondary and tertiary treatment outcomes.

The patient was asymptomatic during the follow-up with no pain, swelling, no relapse as well as a reduction in periapical lesion is observed. The post-operative CBCT images show a reduction in volume of periapical lesion by 86% axially and 78% sagittally (CBCT-PAI score-4D), measured using a Volux–Horos viewer for Mac (V2.0.2). The root length and canal dentin thickness increased by 1.1 times and 1.2 to 1.8 times, respectively. In a study by Jeeruphan et al., 100% resolution of the disease process was observed with REP in comparison to apexification (77–95%). The average root length and root thickness increase observed by them was 14.9% and 28.2%, respectively [27]. However, recently, a retrospective study has shown that the degree of root development after REP is unpredictable due to various factors [28].

The two interesting observations in this case report are that there was mineralized deposition below the Biodentine™ coronal barrier and response to pulp vitality tests even with partially resolved periapical lesion. Similar mineralization below the coronal seal has been reported in cases of successful revitalization and could attributed to the material’s ability of inducing differentiation of stem cells in vivo [26]. However, it was observed in CBCT that the mineralization was seen only from the buccal aspect and not completely over the Biodentine™ seal (Figure 2d). A positive pulp vitality test is observed in 50% of REP treated cases. Specific chemical signals might guide the free nerve endings of the primary afferents into the canal, which is detected by stimulating the functional nociceptors. The mechanism by which these newly recruited neurons respond is still unclear but suggests the presence of vital tissue in the root canal [26]. In our case, vitality is observed even when the periapical lesion is still healing. A recent study has shown that, among clinically successful cases, 46% of REP showed an incomplete root development with a wide open apex observed over 12 to 45 months of follow up. Those roots that appear normal and mature after REP in conventional radiographs have been shown to have irregular canal shapes in CBCT [29].

## 4. Conclusions

HAM, due to its antifibrotic property, is considered to favor regeneration, but the nature of tissue that is formed inside the canal can be confirmed only by histologic assessment. In our case, whether regeneration has been achieved instead of guided endodontic repair has not been confirmed. HAM, when used as a scaffold, has promoted the healing of lesion, thickening of root canal dentin, as well as reestablishment of vital tissue, which constitutes success from a patient and clinician perspective. Further histological studies are required for assessing the histological success of this material as a scaffold in REP.
